# Integrated mechanisms linking sodium–potassium imbalance to salt‐sensitive hypertension

**DOI:** 10.14814/phy2.70715

**Published:** 2025-12-25

**Authors:** Chileleko Siakabanze, Emmanuel Luwaya, Lweendo Muchaili, Lukundo Siame, Frederick Sibbenga, Macrichard Tande, Chikwaniso Shawa, Sepiso K. Masenga, Situmbeko Liweleya

**Affiliations:** ^1^ HAND Research Group, School of Medicine and Health Sciences Mulungushi University, Livingstone Campus Livingstone Zambia; ^2^ Division of Integrated Science Livingstone Center for Prevention and Translational Science Livingstone Zambia

**Keywords:** blood pressure, homeostasis, hypertension, pathogenesis, potassium, salt intake, salt sensitivity, sodium

## Abstract

Salt‐sensitive hypertension (SS‐HT) represents a clinically heterogeneous and mechanistically distinct phenotype of blood pressure dysregulation in which sodium intake disproportionately elevates blood pressure. SS‐HT affects up to 50%–60% of individuals with hypertension globally, with an even greater burden among individuals of African ancestry, postmenopausal women, and those with obesity or metabolic syndrome. SS‐HT arises from multifactorial dysregulation of renal, vascular, and immune systems. Central to its pathophysiology is aberrant activation of the epithelial sodium channel (ENaC), which drives sodium reabsorption in the distal nephron. ENaC activity is enhanced by both aldosterone‐dependent and ‐independent mechanisms. Concurrently, high dietary sodium induces oxidative stress through NADPH oxidase–mediated reactive oxygen species (ROS) production, disrupts nitric oxide (NO) signaling, and activates antigen‐presenting dendritic cells, triggering T‐cell–mediated vascular and renal inflammation. This review proposes a systems‐level framework in which SS‐HT reflects the convergence of ENaC hyperactivation, immunometabolic priming, and hormonal modulation, shaped by sex, race, and dietary sodium–potassium imbalances. Understanding SS‐HT as a multifaceted systems disorder opens new avenues for personalized prevention and treatment. Population‐specific interventions, such as ENaC‐targeting therapies, potassium‐enriched diets, and sex and ancestry‐informed modulation of the renin–angiotensin–aldosterone system (RAAS), represent promising strategies for precision medicine.

## 1 INTRODUCTION

Salt‐sensitive hypertension (SS‐HT) is a growing global health issue, significantly contributing to morbidity and mortality from cardiovascular, renal, and metabolic disorders (Choi et al., 2015; Masenga et al., 2025). Excessive salt intake, especially when coupled with genetic predisposition, alters vascular tone and renal sodium handling, exacerbating blood pressure (BP) elevations (Farquhar et al., 2015). A unifying hypothesis proposes that SS‐HT is a systemic condition arising from ENaC hyperactivation, immune cell sodium‐loading, and sex hormone‐driven vascular and renal modulation (Ahmad et al., 2023). This review explores the multifactorial mechanisms, focusing on ENaC's interaction with immune and hormonal systems and identifies pathways amenable to precision‐targeted interventions.

Excessive dietary salt consumption is considered the most important lifestyle factor that increases blood pressure (BP) and potentiates the development of salt‐sensitive hypertension (SS‐HT), a major public health concern worldwide (Masenga et al., 2023; Nishimoto et al., 2024; Pilic et al., 2016; Rust & Ekmekcioglu, 2017). High salt intake promotes sodium retention, increases fluid volume, and increases vascular tone, consequently an elevation in the BP (Dickinson et al., 2011; Grillo et al., 2019; Thomas & Harvey, 2023). It is a primary contributor to cardiovascular diseases, cerebrovascular accidents, kidney disease, and overall mortality (Bardhan et al., 2024; Mills et al., 2020; Mutengo et al., 2023).

Given the high global prevalence of hypertension (HTN) and its associated morbidity and mortality (Ataklte et al., 2015; Grillo et al., 2019; Mills et al., 2020; Muchaili et al., 2025; Whelton, 2015), elucidating the mechanistic links between salt intake, blood pressure regulation, and salt sensitivity is critical for the development of targeted interventions against salt‐sensitive hypertension (SS‐HT) (Luzardo et al., 2015; Masenga et al., 2024; Rust & Ekmekcioglu, 2017). SS‐HT represents a phenotype in which blood pressure (BP) is disproportionately elevated in response to dietary sodium, contributing to a substantial proportion of the global hypertensive population.

Epidemiological estimates indicate that SS‐HT affects approximately 25% of normotensive individuals and 50–60% of those with established hypertension (Balafa & Kalaitzidis, 2021). Overall, global HTN prevalence ranges between 28% and 55% in both younger and older adults (Masenga, Pilic, Hamooya, et al., 2022; Monakali et al., 2018; Ware et al., 2017). In the United States alone, approximately 43 million individuals, about 24% of the adult population—are affected by hypertension (Fryar & Zhang, 2017; McMaster et al., 2015). In sub‐Saharan Africa, prevalence rates are estimated at 34%, often exacerbated by poor adherence to salt reduction strategies despite clear dietary recommendations from the World Health Organization (WHO) and American Heart Association (AHA) (Fryar & Zhang, 2017; McMaster et al., 2015), which advise limiting daily salt intake to ≤5 g (approximately 2300 mg or 100 mmol of sodium) (Aggarwal et al., 2021). Nevertheless, average daily salt consumption far exceeds these thresholds, reaching approximately 9 g in the United States and up to 12 g in many African settings (Aggarwal et al., 2021).

Salt sensitivity of blood pressure (SSBP) refers to the trait wherein BP changes proportionally with variations in sodium intake (Masenga, Pilic, Hamooya, et al., 2022; Yang et al., 2007). This phenotype is more commonly observed in elderly individuals, females across all age groups, individuals of African descent, those with obesity, and people with metabolic syndrome (Rust & Ekmekcioglu, 2017). Genetic factors also contribute, with polymorphisms, particularly in cytochrome P450 enzymes, modulating individual responses to sodium intake (Maaliki et al., 2022; Sonuch et al., 2021).

SS‐HT is associated with a cascade of deleterious outcomes, including microvascular inflammation, endothelial dysfunction, vascular remodeling, and impaired renal function (Masenga et al., 2019). Clinically, it significantly increases the risk of myocardial infarction, stroke, heart failure, renal failure, and premature mortality, posing a substantial global healthcare burden (Masenga et al., 2019). Despite these profound consequences, the precise molecular mechanisms underlying SS‐HT remain incompletely understood. This underscores the need for an integrative review that synthesizes emerging evidence on the immunological, hormonal, and renal contributors to salt sensitivity to inform future therapeutic strategies.

## 2 SODIUM AND POTASSIUM RATIO ON BLOOD PRESSURE

The World Health Organization recommends an adult dietary sodium intake of less than 2 g/day and potassium intake greater than 3.5 g/day, translating to an optimal Na^+^/K^+^ ratio of ≤0.6 (Masenga et al., 2025). Epidemiological and interventional studies have consistently shown that a lower Na^+^/K^+^ ratio correlates with lower blood pressure and improved cardiovascular outcomes (Jung et al., 2011; Strazzullo et al., 2009). High sodium intake elevates blood pressure through mechanisms such as intravascular volume expansion (Whelton, 2015), increased cardiac output, heightened peripheral vascular resistance, and impaired renal sodium excretion, all of which are amplified in individuals with SS‐HT (Baldo et al., 2015; Elijovich et al., 2020).

Recent evidence reveals that sodium distribution in the body is more complex than previously appreciated. Beyond conventional compartments such as plasma and interstitial fluid, sodium can accumulate in osmotically inactive reservoirs, particularly in skin and skeletal muscle, under hypertonic conditions (Elijovich et al., 2020; Nijst et al., 2015). This non‐osmotic sodium storage has been implicated in the activation of immune cells, specifically dendritic cells, which in turn prime T lymphocytes and promote their infiltration into renal and vascular tissues (Mian et al., 2014; Norlander et al., 2018). These immune‐mediated responses facilitate cytokine release, including TNF‐α, IL‐17, and IL‐10, driving endothelial dysfunction, renal injury, and sustained blood pressure elevation (Mian et al., 2014; Norlander et al., 2018).

Conversely, dietary potassium exerts antihypertensive effects through multiple mechanisms. Potassium facilitates natriuresis, reduces sympathetic vasoconstriction, and improves endothelial‐dependent vasodilation by enhancing nitric oxide bioavailability (Little et al., 2023; Nomura et al., 2019; van der Wijst et al., 2018; World Health Organization, 2012). Furthermore, potassium promotes baroreceptor sensitivity, attenuates oxidative stress, and modulates insulin responsiveness and vascular smooth muscle tone (An et al., 2022). Approximately 90% of ingested potassium is excreted renally, underscoring the kidney's central role in maintaining potassium homeostasis and its interactions with sodium handling (Amador et al., 2014; Stone et al., 2016).

The American Heart Association (AHA) recommends a sodium intake below 2300 mg/day, with an ideal target of 1500 mg/day for individuals with hypertension, diabetes, or chronic kidney disease. Simultaneously, potassium intake of at least 4700 mg/day from dietary sources such as fruits, vegetables, and dairy is advised to optimize cardiovascular protection (Amador et al., 2014; Stone et al., 2016). Unfortunately, most global populations fail to meet potassium intake recommendations while exceeding sodium guidelines, creating an unfavorable Na^+^/K^+^ ratio that contributes to widespread hypertension.

At the molecular level, the distal convoluted tubule (DCT) of the kidney plays a pivotal role in sodium and potassium balance via the sodium‐chloride cotransporter (NCC). High sodium intake prompts a physiological adaptation in the kidney characterized by a reduction in the activity of the NCC (Borrelli et al., 2020). This diminished function serves as a compensatory mechanism to facilitate the excretion of the excess sodium, hence maintaining stable blood pressure and fluid volume (Hamm et al., 2010; Strazzullo et al., 2009). To the contrary, dietary sodium restriction triggers the opposite response, leading to an upregulation of NCC activity to promote sodium conservation (Pratt, 2005; Vallon et al., 2009). This dietary modulation of ion transport also influences calcium and magnesium handling in the DCT, linking micronutrient status to hypertensive risk (Schmidlin et al., 1999). Additionally, genetic variations in renal ion transporters and regulatory pathways, including those involving WNK kinases, NCC, and ENaC, contribute to individual differences in sodium retention and salt sensitivity (Schmidlin et al., 1999).

In postmenopausal women, declining estrogen levels disrupt the balance between nitric oxide and angiotensin II signaling, leading to increased RAAS activation, oxidative stress, and altered sodium handling. These changes collectively promote vascular dysfunction and elevate hypertensive risk in this population (Hernandez Schulman & Raij, 2006; Kelly et al., 2013; White et al., 1997). As such, sodium‐potassium balance and renal handling are not only central to blood pressure regulation but are also influenced by age, sex hormones, and genomic factors, reinforcing the need for personalized dietary and pharmacologic interventions in SS‐HT (Kelly et al., 2013).

## 3 MECHANISTIC ROLE OF EPITHELIAL Na^+^ CHANNEL IN SALT‐SENSITIVE HYPERTENSION

A key downstream effector of sodium‐potassium homeostasis is the epithelial sodium channel (ENaC), which plays a pivotal mechanistic role in the pathogenesis of salt‐sensitive hypertension. ENaC functions as a critical regulator of renal sodium reabsorption and fluid balance in the distal nephron, particularly within the connecting tubule and collecting duct, thereby exerting a significant influence on blood pressure homeostasis (Mutchler et al., 2021). Structurally, the canonical ENaC is composed of three subunits, alpha (α), beta (β), and gamma (γ), while in extrarenal tissues, a delta (δ) subunit may substitute for α to form functional heterotrimeric channels (Ahmad et al., 2023; Padmanabhan et al., 2012). Each subunit contains an extracellular domain that detects luminal sodium levels, two transmembrane‐spanning helices forming the ion‐conducting pore, and cytoplasmic termini that mediate regulatory protein interactions and post‐translational modifications (Demirci et al., 2024; Jia et al., 2018; Sowers et al., 2019).

The mechanistic relevance of ENaC in SS‐HT has been demonstrated across various models. In Dahl salt‐sensitive (Dahl‐SS) rats, a prototypical model of sodium‐sensitive hypertension, ENaC expression and activity are markedly upregulated upon high salt intake, leading to early and sustained elevations in blood pressure (Lang & Pearce, 2016; Sun et al., 2011). Conversely, salt‐resistant rats exhibit attenuated ENaC responses accompanied by lower aldosterone levels. Pharmacologic blockade of ENaC using amiloride or benzamil has been shown to blunt hypertensive responses in Dahl‐SS rats, supporting a causal role for ENaC hyperactivity in sodium‐driven blood pressure elevation (Lang & Pearce, 2016; Sun et al., 2011). Amiloride inhibits ENaC‐mediated sodium transport, thereby promoting natriuresis and potassium conservation, whereas benzamil acts via a similar mechanism but alters calcium fluxes instead of potassium retention (Lang & Pearce, 2016; Sun et al., 2011).

Notably, ENaC overactivity in Dahl‐SS rats occurs despite suppressed renin‐angiotensin‐aldosterone system (RAAS) activity, suggesting aldosterone‐independent activation. This phenomenon has been linked to aberrant activation of the Rac1 GTPase, which stimulates serum‐ and glucocorticoid‐regulated kinase 1 (SGK1), a downstream effector of the mineralocorticoid receptor (MR) pathway that enhances ENaC surface expression (Lang & Pearce, 2016; Sun et al., 2011). Rac1 also serves as a critical component of NADPH oxidase (NOX) complexes, particularly NOX4, which generates reactive oxygen species (ROS). ROS, in turn, modulate ENaC activity via prostaglandin or PI3K‐dependent pathways, establishing a feedforward loop between sodium retention and oxidative stress (Pitzer et al., 2020).

Proteolytic cleavage represents another vital mechanism for ENaC activation. The α‐ and γ‐subunits are sequentially cleaved by intracellular furin within the Golgi and subsequently by extracellular serine proteases such as prostasin, plasmin, elastase, kallikrein, and matriptase, facilitating full channel activation (Aufy et al., 2023). Enhanced proteolytic processing of ENaC has been documented in salt‐sensitive rodents fed high‐salt diets and correlates with elevated blood pressure. Pharmacological inhibition of these proteases using camostat mesylate reduces blood pressure, highlighting the therapeutic relevance of proteolytic regulation (Rivera et al., 2017).

In addition to proteolysis, ENaC function is subject to fine‐tuning through post‐translational modifications, including phosphorylation and palmitoylation, and through feedback regulation by intracellular sodium concentrations (Rivera et al., 2017). Exogenous aldosterone administration has been shown to further enhance ENaC cleavage and activity, reinforcing the role of hormonal modulation (Rivera et al., 2017). Although ENaC is central to SS‐HT pathogenesis, it does not act in isolation. Other molecular systems, including the Na^+^/K^+^ ATPase, NCC, immune‐inflammatory pathways, nitric oxide signaling, and mTOR‐regulated metabolic circuits, may converge to modulate sodium handling and vascular tone in salt‐sensitive individuals (Pavlov & Staruschenko, 2017; Vieira‐Filho et al., 2014).

Chronic kidney disease (CKD) is a clinical model of salt sensitivity where impaired sodium excretion exaggerates blood pressure responses to salt intake (Borrelli et al., 2020). Progressive nephron loss limits pressure natriuresis and enhances extracellular volume expansion (Ellison, 2017). In this setting, ENaC and the sodium–chloride cotransporter (NCC) become hyperactive, promoting sodium retention and hypertension despite reduced glomerular filtration. RAAS overactivation and increased aldosterone levels further upregulate ENaC expression, worsening sodium‐dependent hypertension (Pavlov & Staruschenko, 2017). Uremic toxins, oxidative stress, and inflammation also impair nitric oxide signaling, increasing vascular stiffness and endothelial dysfunction (Harlacher et al., 2022). Clinical studies confirm that moderate sodium restriction in CKD lowers blood pressure, reduces proteinuria, and slows kidney function decline. Therefore, CKD exemplifies how renal dysfunction, ENaC dysregulation, and inflammatory activation converge to drive salt‐sensitive hypertension.

## 4 LIDDLE SYNDROME AND ITS RELEVANCE TO SALT‐SENSITIVE HYPERTENSION

Liddle syndrome provides a monogenic model for understanding how ENaC hyperactivity drives salt‐sensitive hypertension (Mubarik et al., 2025). It results from gain‐of‐function mutations in the Sodium Channel Non‐Neuronal Subunit (SCNN1) alpha, SCNN1beta, or SCNN1gamma genes encoding ENaC subunits (Chen et al., 2013). These mutations prevent normal ubiquitination and degradation of ENaC, causing persistent sodium reabsorption in the distal nephron. The resulting sodium retention leads to plasma volume expansion, low plasma renin activity, and suppressed aldosterone secretion. Despite low aldosterone, hypertension in Liddle syndrome remains severe and salt dependent, highlighting the critical role of ENaC overactivity in blood pressure regulation (Bubien, 2010). Insights from Liddle syndrome therefore support the broader mechanistic framework in which aberrant ENaC activation, whether genetic, hormonal, or inflammatory, forms a final common pathway for salt‐sensitive hypertension.

## 5 RACIAL AND SEX DIFFERENCES IN SALT SENSITIVITY AND BLOOD PRESSURE

Emerging evidence underscores the multifactorial and intersectional nature of salt‐sensitive hypertension (SS‐HT), with race and sex serving as major axes of variability in both susceptibility and clinical manifestation. Epidemiological data from the United States indicate that African American populations have the highest prevalence of hypertension and related cardiovascular disease (CVD) morbidity and mortality when compared to other racial groups (Ahmed & Layton, 2020; Jeong et al., 2024). Importantly, race is not a fixed biological or genetic construct but a social category with profound implications for health via differential exposures, stressors, healthcare access, and dietary patterns (Brothers et al., 2019; Bunsawat et al., 2022). As such, the pathobiology of SS‐HT in racially diverse populations likely reflects a confluence of genetic predispositions, environmental modifiers, social determinants of health (SDoH), and epigenetic mechanisms (Faulkner & Belin de Chantemèle, 2020; Shukri et al., 2018).

Inflammation and oxidative stress appear to disproportionately affect racially minoritized groups. Vascular inflammation, marked by endothelial activation, leukocyte infiltration, and release of pro‐inflammatory cytokines (e.g., IL‐6 and TNF‐α), drives increased vascular tone and arterial stiffness, thereby exacerbating hypertensive risk (Strazzullo et al., 2009). This is further compounded by elevated levels of reactive oxygen species (ROS), which disrupt nitric oxide (NO) bioavailability, impair vasodilation, and potentiate vascular dysfunction (Tran et al., 2020; Zhang, Yuan, et al., 2022). Excessive sodium intake amplifies this inflammatory milieu, contributing to systemic endothelial damage and impaired autoregulation in both macro‐ and microvasculature (Tran et al., 2020; Zhang, Yuan, et al., 2022).

In parallel, neurohormonal regulatory systems, including the sympathetic nervous system and renin‐angiotensin‐aldosterone system (RAAS), mediate rapid and long‐term blood pressure control. Sympathetic activation via the baroreceptor reflex enhances cardiac output and vascular tone in response to hypotension (Gardner et al., 2015; Parksook et al., 2022). RAAS, meanwhile, modulates intravascular volume and systemic resistance via sequential release of renin, angiotensin II (Ang II), and aldosterone (Pereira‐Acácio et al., 2022). Dysregulation of this axis, especially in the context of sodium excess, leads to sodium retention, increased peripheral resistance, and sustained hypertension (De Giusti et al., 2013; Graudal et al., 2017; Hilliard et al., 2013; Mill et al., 2019). Paradoxically, in SS‐HT individuals, high sodium intake fails to suppress RAAS activity adequately, highlighting a pathophysiological hallmark of this condition (Hilliard et al., 2013; Mill et al., 2019).

Sex‐specific differences further modulate SS‐HT risk. In women, especially premenopausal individuals, the RAAS exhibits a cardioprotective phenotype, characterized by increased expression of ACE2, Ang (1–7), Mas receptor (MasR), and AT_2_ receptors. These pathways promote vasodilation, NO release, and anti‐inflammatory signaling (Kanashiro‐Takeuchi et al., 2009). In contrast, males demonstrate a dominance of the classical RAAS arm, ACE/Ang II/AT1 receptor, which promotes vasoconstriction and sympathetic outflow. However, postmenopausal women experience a shift toward this maladaptive axis due to estrogen deficiency, resulting in heightened salt sensitivity and BP dysregulation (Faulkner et al., 2020; Yeo et al., 2024). Interestingly, despite lower systemic Ang II levels, women exhibit greater aldosterone responses, contributing to impaired natriuresis and increased sodium retention (Yeo et al., 2024).

**FIGURE 1:**
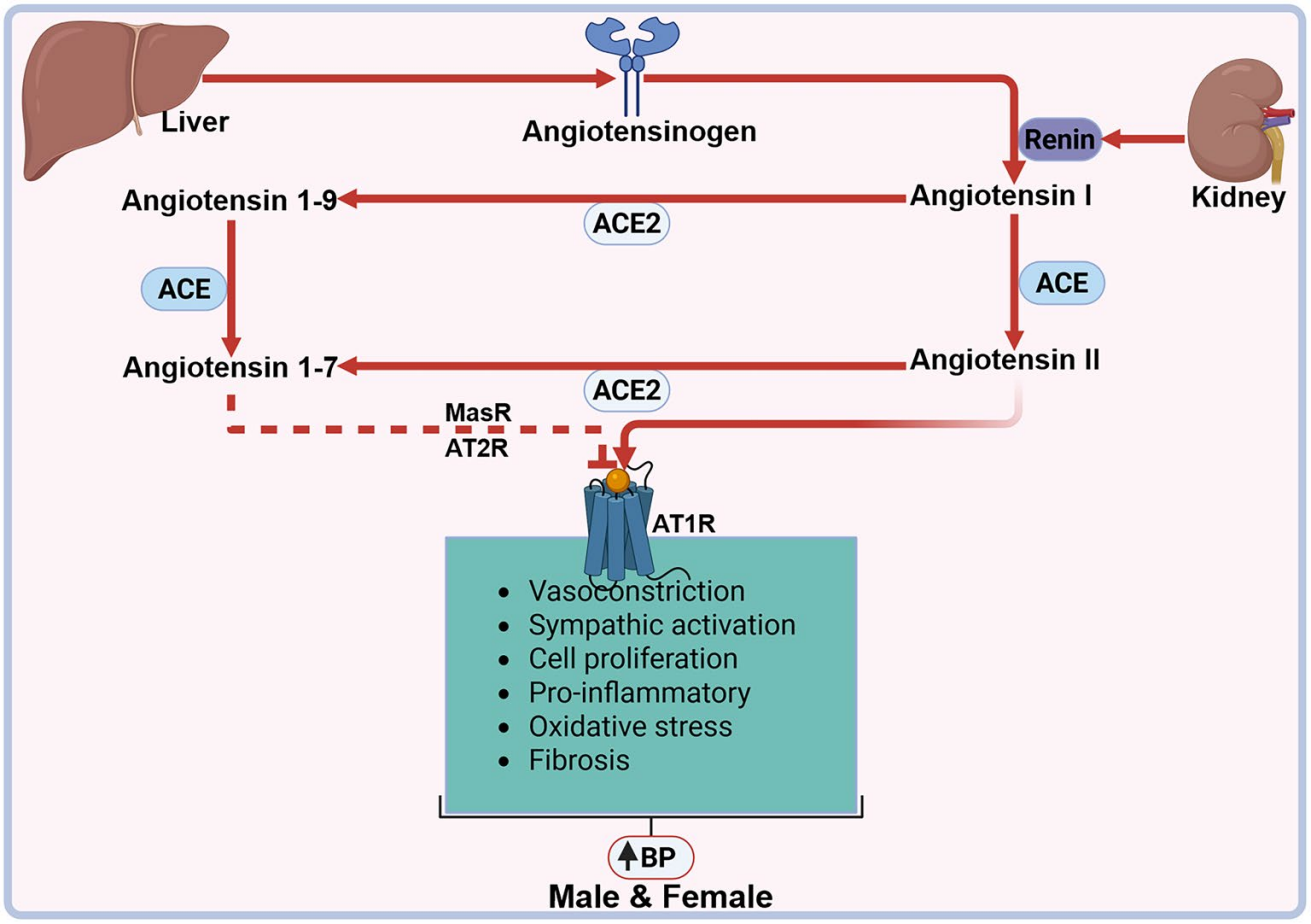
The renin–angiotensin–aldosterone system (RAAS) regulates cardiovascular and renal function through a cascade of hormones. Renin, released by juxtaglomerular cells in the kidney, initiates the process by cleaving angiotensinogen to form angiotensin I via the N terminus. This peptide is subsequently converted by ACE into angiotensin II, the system's primary active agent. Angiotensin II exerts its effects primarily by binding to two receptors: type 1 (AT1R) and type 2 (AT2R). Activation of AT1R, leads to vasoconstriction, vascular remodeling, and renal fibrosis. Additionally, angiotensin II stimulates aldosterone secretion from the adrenal cortex, a hormone critical for sodium and water retention, ultimately causing an elevation in the BP. AT2R and MasR have cardioprotective effects, however in SS‐HT their effects are overridden by AT1R influences hence sustaining an elevation in the BP. ACE, angiotensin‐converting enzyme; ACE2, angiotensin‐converting enzyme 2; AT1R, angiotensin II receptor type 1; AT2R, angiotensin II receptor type 2; BP, blood pressure. Created in BioRender. Masenga, S. (2025) https://BioRender.com/fu7czsy.

Population‐level and experimental data support the assertion that salt sensitivity is clinically more prevalent in females than males (Bailey & Dhaun, 2024; Faulkner et al., 2018; Mary et al., 2022). Women display larger BP responses to sodium loading and higher frequencies of sodium‐sensitive hypertension. Behavioral and physiological mechanisms may converge here, with animal studies suggesting androgen‐mediated suppression of sodium appetite in males and heightened sodium preference in females (Mary et al., 2022). These differences, amplified by sex hormones, receptor‐level divergence, and sociocultural determinants, underscore the need for sex‐ and race‐specific approaches to managing salt‐sensitive hypertension (Mary et al., 2022). A systems biology model that incorporates hormonal, immune, and environmental inputs will be pivotal to advancing personalized interventions (Mary et al., 2022). Racial and sex differences may further suggest genetic variability to condition predisposition. Genome‐wide association studies have identified common loci that modify blood pressure and salt sensitivity (Wang & Wang, 2019). In a study conducted by Elijovich et al. (2024), they revealed that variants in the uromodulin gene (UMOD) associate with salt sensitivity, hypertension, and altered renal sodium handling. Large sequencing studies have further found rare ENaC (SCNN1A/SCNN1B/SCNN1G) variants that exert large effects on blood pressure (Blobner et al., 2022). See Figure 1.

### 5.1 Racial differences in vascular inflammation and oxidative stress

Many studies have found that Black individuals' vascular function is lower than that of White adults (Blobner et al., 2022). A study of young male participants found that young Black individuals had thicker carotid artery walls, reduced arterial elasticity, and elevated aortic stiffness compared to their White counterparts (Mulamfu et al., 2025). Additionally, they exhibited reduced peripheral blood circulation and higher central blood pressure, despite displaying similar peripheral (brachial) blood pressure readings (Barris et al., 2023; Elijovich et al., 2024; Sahinoz et al., 2021). Two new studies utilizing gold‐standard techniques found ethnic disparities in vascular function in healthy young female volunteers, adding to an already extensive body of research (Sahinoz et al., 2021). In particular, as compared to White participants, Black participants showed poorer microvascular function (Sahinoz et al., 2021). A combination of in vitro and in vivo evidence indicates that Black Americans have higher levels of oxidative stress and vascular inflammation than white Americans (Kurtz et al., 2021). Lower levels of glutathione, an antioxidant biomarker, are connected to endothelial dysfunction (Zhang, Pan, et al., 2022). In terms of inflammation, higher levels of C‐reactive protein (CRP) are associated with an increased risk of hypertension (Zhang, Pan, et al., 2022). Studies have shown that black individuals have higher circulating CRP levels than white adults. Nonetheless, many studies have under‐represented racial and ethnic minorities (Zhang, Pan, et al., 2022).

### 5.2 Ethnic variations in renal physiology and circulatory resistance

Research findings have been inconsistent, but some data suggest that Black individuals exhibit reduced plasma renin activity and aldosterone concentrations relative to other ethnic groups (Kim et al., 2024). This observation holds potential clinical relevance, as the physiological adaptation to elevated sodium intake normally involves suppression of RAAS components, causing downregulation of the epithelial sodium channel (ENaC) function and enhancing urinary sodium elimination, counterbalancing excess dietary sodium retention (Pitzer et al., 2022). Interestingly, potassium supplementation has been shown to enhance RAAS activity in Black people, potentially improving sodium excretion and blood pressure regulation (Shukri et al., 2018). Emerging studies indicate that potassium intake in sodium‐sensitive African American individuals may counteract the renal hemodynamic effects of high sodium consumption by preserving kidney blood flow and reducing sodium‐triggered rises in vascular resistance and arterial pressure (Shukri et al., 2018).

SSBP is caused by the interplay of several complex systems, including biological and environmental influences (Kawarazaki et al., 2020; Jones & Rayner, 2021; Robinson et al., 2019). The pathophysiological basis for SS‐HT involves interconnected pathways such as vascular oxidative damage and inflammatory activation, dysregulation of renal hemodynamics and circulatory tone, alterations in intestinal microbial ecology, socioeconomic and environmental influences, and lifestyle factors including dietary patterns and fluid management (Abais et al., 2014; Pilic et al., 2016; Vogt et al., 2023). Importantly, race/ethnicity is a crucial aspect to consider when studying the pathophysiology of salt‐sensitive blood pressure (Vogt et al., 2023).

## 6 EFFECTS OF SALT ON BLOOD VESSELS, BLOOD PRESSURE AND SSBP

Excessive salt consumption is a major factor in the emergence of hypertension and cardiovascular diseases (CVDs) (Graudal et al., 2017; Liu et al., 2015). Dysregulated sodium handling may impair renal sodium excretion capacity and vascular endothelial integrity (Boero et al., 2002), while stimulating sympathetic nervous activity, RAAS signaling, and endocrine pathways (androgen signaling and insulin dysregulation) (Boero et al., 2002). These interconnected disruptions promote inflammatory responses and oxidative damage (Ertuglu et al., 2022; Kirabo et al., 2014) collectively leading to the development of hypertension (Kirabo et al., 2014).

Human and animal studies have established the link between inflammatory pathways and the pathogenesis of HTN: inflammatory pathways promote oxidative stress, endothelial dysfunction, and immune cell activation (Kirabo et al., 2014; Zhang, Pan, et al., 2022). Inflammation in dendritic cells causes oxidative stress, which promotes IsoLevuglandins (IsoLGs) formation, T cell activation, and hypertension due to massive endothelial injury, vasculopathies, and endothelial dysfunction (Mwape et al., 2024). IsoLGs, reactive derivatives generated through oxidative lipid breakdown, chemically attach to lysine residues in proteins, triggering structural and functional changes to these proteins after their initial synthesis (Mwape et al., 2012). Moreover, protein alteration by IsoLGs may result in neoepitopes that are not recognized as self and promote an immune response mimicking an autoimmune reaction causing hypertension (Xiao et al., 2018). This mechanism may explain why SS‐HT is strongly associated with autoimmune diseases and is more prevalent in females, as autoimmune disorders disproportionately affect women (Xiao et al., 2018).

In addition, experimental studies in mice demonstrate that high sodium concentrations not only elevate blood pressure but also markedly enhance the production of IsoLG‐modified protein complexes within dendritic cells, linking sodium excess to oxidative stress‐related molecular changes in immune cells (Wu et al., 2013; Xiao et al., 2018). Sodium ions (Na^+^) are transported into dendritic cells (DCs) via amiloride‐inhibitable transport mechanisms, followed by their exchange with calcium ions (Ca^2+^) through the sodium‐calcium exchanger (NCX) pathway (McMaster et al., 2015). The entry of Ca^2+^ activates protein kinase C (PKC), consequently phosphorylating the NADPH oxidase subunit p47phox, which leads to NADPH oxidase activation (Abais et al., 2015; Muller et al., 2000), increased superoxide (O2·^−^), derivative reactive oxygen species (ROS) production, and IsoLG formation (Lu & Crowley, 2018). Furthermore, angiotensin II (Ang II) is a powerful stimulator of ET‐1 in vascular smooth muscle and endothelial cells, resulting in the induction of vascular hypertrophy (Bevan, 1993). This mechanism highly supports the development of SS‐HT in males who tend to have poor Ang II antagonistic mediators, such as NO, which is more abundant in females due to increased estrogen levels (Mishra et al., 2019).

High salt consumption is associated with glycocalyx destruction, leading to HTN (Oberleithner, 2015). The glycocalyx is a dense sugar, lipid and protein‐composed layer, whose core function is to maintain blood flow and the health of the endothelium (Masenga, Pilic, Malumani, & Hamooya, 2022). Physiologically, both the glycocalyx and erythrocytes are negatively charged, hence, they repel one another, preventing friction (Masenga, Pilic, Malumani, & Hamooya, 2022). Elevated sodium levels can reduce their buffering capacities, resulting in friction between erythrocytes and the glycocalyx consequently glycocalyx erosion, endothelial cell stimulation, combined with sodium ion migration into perivascular tissue spaces, initiates a sequential immune‐inflammatory response (Melander et al., 2007).

**FIGURE 2:**
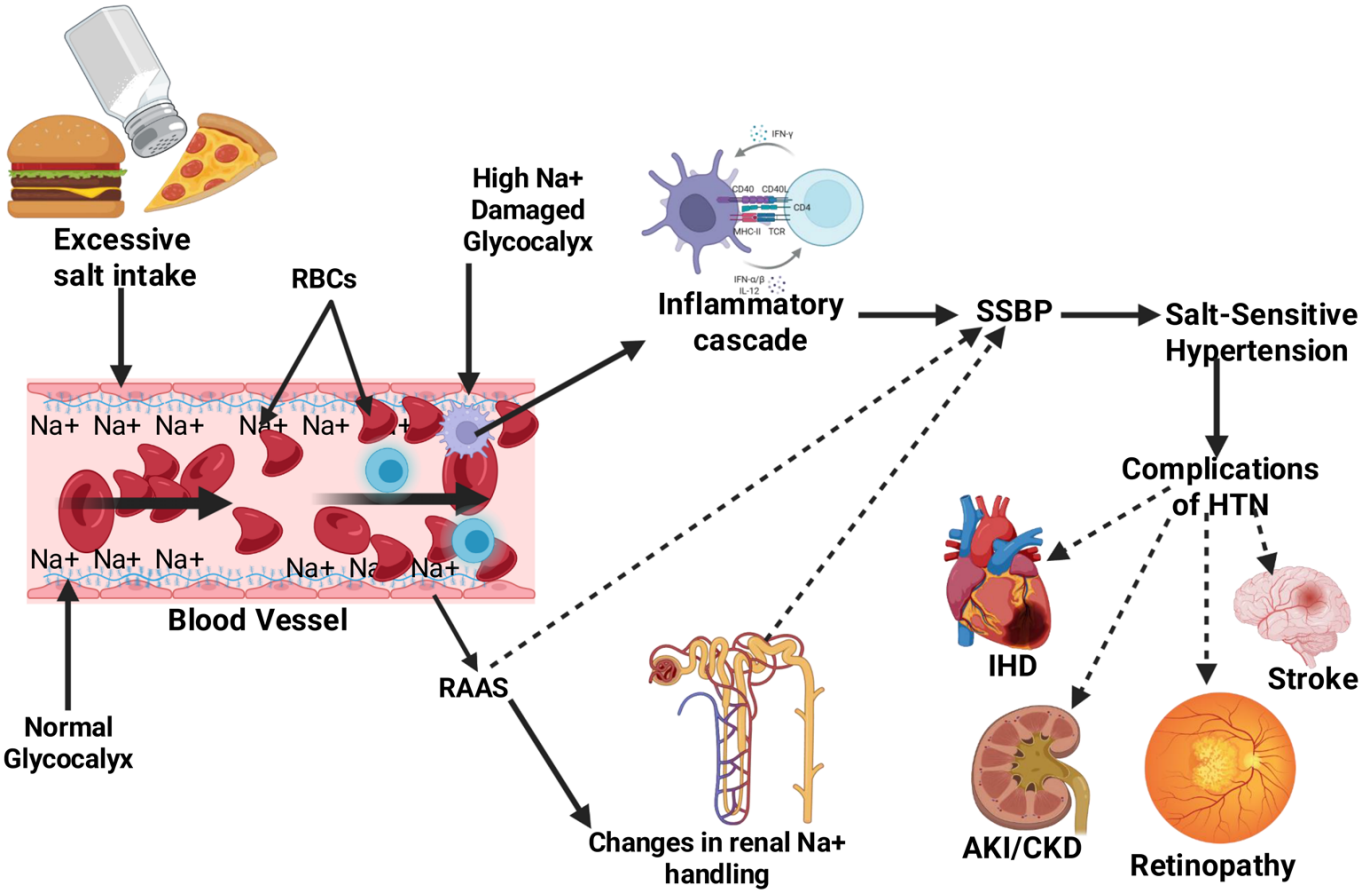
Mechanistic effects of salt on blood vessels, blood pressure, and SSBP. It shows the effects of salt on blood vessels—High salt intake results in negatively charged erythrocytes and the glycocalyx, which serves as a vessel's sodium buffer to have friction, thereby causing damage to the endothelium, activating it, and causing inflammation. In addition, in postmenopausal women, reduction in estrogen, which promotes the production of NO, an antagonist of angiotensin II reduces results to changes in sodium handling, promoting hypertension development. These are the mechanism by which salt‐sensitive blood pressure and hypertension develops, which in turn result in multiple complications, including myocardial infarction, stroke, congestive cardiac failure, retinopathy, and overall mortality. AKI, acute kidney injury; CKI, chronic kidney injury; HTN, hypertension; IHD, ischaemic heart disease; Na^+^, sodium; RAAS, renin–angiotensin–aldosterone system; RBCs, red blood cells; SSBP, salt sensitivity of blood pressure. Created in BioRender. Masenga et al. (2025) https://BioRender.com/yyexn85.

Emerging data highlight the pivotal contribution of immune‐mediated inflammatory pathways to the initiation and progression of hypertension by promoting oxidative stress, endothelial dysfunction, and immune activation (Melander et al., 2007). The accumulation of T cells and macrophages in the kidneys and vasculature leads to vasculopathies contributing to the end‐organ damage that accompanies this disease (Melander et al., 2007). See Figure 2.

## 7 KNOWLEDGE GAPS AND FUTURE PERSPECTIVES

Despite extensive research on SSBP, the mechanisms underlying its development remain elusive. Suffice it to say SSBP has been studied for many years now, however, keynote mechanistic interlinkages among sex differences, immune activation‐inflammation, RAAS activation and its defects and racial discrepancies require much more studies to understand. We recommend an interdisciplinary approach to elucidate the causes of SSBP. Sex differences in RAAS and ENaC functionality in salt‐sensitive hypertension require further exploration. Studies are suggestive of significant racial differences, with Blacks being more susceptible to developing SSBP and tending to have a boost in their RAAS mechanisms upon consumption of dietary K^+^ supplements, mechanisms propagating this phenomenon require further exploration. Furthermore, we recommend dietary modifications, however, dietary interventions alone may not be sufficient for some individuals; other lifestyle modifications and/or therapeutic measures may be necessary to effectively manage salt‐sensitive hypertension.

## 8 CONCLUSION

Excessive salt consumption is a fundamental environmental factor that causes SSBP. However, multiple interlinking mechanisms contribute to the development of SSBP and SS‐HT. Racial and sex differences have been observed in SS‐HT; Blacks and female individuals of all ages are generally more salt sensitive than whites and male individuals of the same age. In women, postmenopausal states due to lower levels of estrogen increase the risk and severity. Studies suggest that Blacks have increased oxidative stress and inflammation compared to other races.

ENaC facilitates electrogenic sodium reabsorption in the aldosterone‐sensitive distal nephron, an action that promotes sodium retention and is physiologically antagonistic to natriuresis (Stockand, 2012). In contrast, ENaC expressed in vascular endothelial cells contributes to endothelial dysfunction through distinct pathophysiological mechanisms (Sternak et al., 2018; Stockand, 2012). Activation of vascular ENaC increases intracellular sodium, which promotes Ca^2+^ influx via the sodium‐calcium exchanger (Hill et al., 2020). This elevated intracellular Ca^2+^ leads to a reduction in NO bioavailability, increased oxidative stress, and a pro‐inflammatory phenotype, resulting in endothelial stiffness and impaired vasodilation (Hill et al., 2020; Sowers et al., 2019).

Salt‐sensitive hypertension is a complex, multifactorial condition involving ENaC dysregulation, immune activation, and hormonal influences. Integrative insights from renal physiology, immunometabolism, and systems biology reveal new targets for intervention. Precision strategies tailored to individual risk profiles, informed by sex, race, and molecular signatures, represent the future of SS‐HT prevention and treatment.

## AUTHOR CONTRIBUTIONS

CS and SL conceptualized the study and wrote the draft manuscript. CS, EL, FS, MT, CS, SKM, and SL wrote and edited different sections of the manuscript. CS created all the figures. CS, EL, FS, LM, MT, LS, CS, and SL edited and reviewed the manuscript. CS and SL conceptualized the framework and finalized the manuscript, as well as obtained funding for the manuscript. All authors contributed to the article review, edited and approved the final version of this manuscript.

## FUNDING INFORMATION

This work did not receive any financial support.

## CONFLICT OF INTEREST STATEMENT

The authors declare no competing interests.

## ETHICS STATEMENT

This manuscript is a review of previously published literature and does not report original research involving human participants or animals. Consequently, ethical approval and informed consent were not required. All studies cited were conducted in accordance with the ethical standards of their respective institutional and national research committees and with the Declaration of Helsinki or comparable ethical guidelines, as appropriate. The authors adhered to principles of academic integrity, appropriate citation, and responsible reporting throughout the preparation of this review.
